# A simulation study on the effects of dendritic morphology on layer V prefrontal pyramidal cell firing behavior

**DOI:** 10.3389/fncel.2014.00287

**Published:** 2014-09-16

**Authors:** Maria Psarrou, Stefanos S. Stefanou, Athanasia Papoutsi, Alexandra Tzilivaki, Vassilis Cutsuridis, Panayiota Poirazi

**Affiliations:** ^1^Institute of Molecular Biology and Biotechnology, Foundation for Research and Technology–HellasHeraklion, Greece; ^2^Centre for Computer Science and Informatics Research, Science and Technology Institute, University of HertfordshireHatfield, UK; ^3^School of Computer Science, University of HertfordshireHatfield, UK; ^4^Department of Biology, University of CreteHeraklion, Greece

**Keywords:** dendrites, PFC, morphology, pyramidal cell, single neuron modeling, firing pattern, layer V

## Abstract

Pyramidal cells, the most abundant neurons in neocortex, exhibit significant structural variability across different brain areas and layers in different species. Moreover, in response to a somatic step current, these cells display a range of firing behaviors, the most common being (1) repetitive action potentials (Regular Spiking—RS), and (2) an initial cluster of 2–5 action potentials with short interspike interval (ISIs) followed by single spikes (Intrinsic Bursting—IB). A correlation between firing behavior and dendritic morphology has recently been reported. In this work we use computational modeling to investigate quantitatively the effects of the basal dendritic tree morphology on the firing behavior of 112 three-dimensional reconstructions of layer V PFC rat pyramidal cells. Particularly, we focus on how different morphological (diameter, total length, volume, and branch number) and passive [Mean Electrotonic Path length (MEP)] features of basal dendritic trees shape somatic firing when the spatial distribution of ionic mechanisms in the basal dendritic trees is uniform or non-uniform. Our results suggest that total length, volume and branch number are the best morphological parameters to discriminate the cells as RS or IB, regardless of the distribution of ionic mechanisms in basal trees. The discriminatory power of total length, volume, and branch number remains high in the presence of different apical dendrites. These results suggest that morphological variations in the basal dendritic trees of layer V pyramidal neurons in the PFC influence their firing patterns in a predictive manner and may in turn influence the information processing capabilities of these neurons.

## Introduction

Cortical neurons exhibit a wide range of firing behaviors (Connors and Gutnick, 1990). Pyramidal cells in particular, the most abundant cortical excitatory neurons, have been shown to fire in at least three different patterns: (a) repetitive action potentials with or without adaptation (Regular Spiking—RS), (b) an initial doublet followed by single spikes (Intrinsic Bursting—IB), or (c) repetitive bursts (2–5 action potentials with inter-spike-intervals of less than 10 ms) (Repetitive Oscillatory Bursting—ROB) (Yang et al., 1996; Dégenètais et al., 2002; Wang et al., 2006; Chang and Luebke, 2007; Van Aerde and Feldmeyer, 2013).

The abovementioned firing patterns are likely to serve distinct functions within the network and contribute differentially to its behavior. Experimental studies have shown that bursts improve the signal-to-noise ratio of neuronal responses and convey specific stimulus-related information (Eggermont and Smith, 1996; Martinez-Conde et al., 2002). In synapses with short-term facilitation, bursts are transmitted more reliably than isolated spikes (Lisman, 1997). Other studies have shown a link between neuronal sub-types, their outputs and their target areas. IB neurons in layer V of the auditory cortex send signals to higher-order thalamic nuclei as well as midbrain and brainstem nuclei, whereas RS neurons send signals to the ipsilateral and contralateral cortex (Sun et al., 2013). Similarly, IB and RS neurons in the prefrontal cortex (PFC) project to the pons (cortico-pontine) or the striatum but no IB neurons project to the contralateral cortex (cortico-cortical) (Morishima and Kawaguchi, 2006). IB neurons in the distal parts of the subiculum project primarily to the medial enthorhinal cortex but not the amygdala (Kim and Spruston, 2012). This segregation seen across several brain areas is likely to be associated with some form of functional specialization of RS and IB neurons. Therefore, it is important to understand what features of their morphology, connectivity and/or biophysics may determine the firing pattern of cortical pyramidal neurons.

Studies have shown that sources of firing pattern variability may be the distribution and density of active mechanisms within a cell (Jensen et al., 1994; Andreasen and Lambert, 1995; Migliore et al., 1995; Jensen and Yaari, 1997; Schwindt and Crill, 1999), synaptic connectivity (Williams and Johnston, 1989; Weisskopf et al., 1994; Nicoll and Malenka, 1995; Maccaferri et al., 1998; Yeckel and Berger, 1998; Sidiropoulou and Poirazi, 2012; Sun et al., 2013), morphological diversity (Bilkey and Schwartzkroin, 1990; Chagnac-Amitai et al., 1990; Mason and Larkman, 1990; Mainen and Sejnowski, 1996; Yang et al., 1996; Krichmar et al., 2002; Van Ooyen et al., 2002; Van Elburg and van Ooyen, 2010), and inhibition on soma and/or dendrites of the cell (Lovett-Barron et al., 2012; Royer et al., 2012).

Neuronal firing behavior depends strongly on the distribution and density of ionic currents. Neurophysiological studies have shown that RS pyramidal cells can generate bursts if the extracellular K^+^ concentration is increased (Jensen et al., 1994; Andreasen and Lambert, 1995; Jensen and Yaari, 1997; Schwindt and Crill, 1999). Similarly, a modeling study showed that the CA3 pyramidal cell's firing characteristics can be changed from non-bursting to bursting by modifying the Ca^2+^-independent K^+^ conductance 100 μm from the soma (Migliore et al., 1995). Along the same line, the modeling study of Sidiropoulou and Poirazi (2012) showed that doubling the Na^+^ and Ca^2+^ conductances turns an RS LV PFC cell into an IB one. Combination of neurophysiology with computational modeling has suggested that pyramidal cell bursting may be due to the interplay of somatic and dendritic voltage-gated Na^+^ and K^+^ conductances (Krahe and Gabbiani, 2004). These conductances promote propagation of action potentials from the soma into the dendrites, causing the dendrites to be depolarized when, at the end of a somatic spike, the soma is hyperpolarized, leading to a rebound current from the dendrites to the soma. This rebound current causes a depolarizing after-potential at the soma, which, if strong enough, may lead to another somatic spike. This whole process has been described as a “ping-pong” interaction between the soma and the dendritic tree (Wang, 1999).

In addition to intrinsic mechanisms, changes in the strength, timing or connectivity of synaptic input can alter the firing behavior of a neuron. Studies have shown that long-term potentiation of mossy fibers to CA3 pyramidal cell synapses may cause post-synaptic pyramidal cells to burst (Williams and Johnston, 1989; Weisskopf et al., 1994; Nicoll and Malenka, 1995; Maccaferri et al., 1998; Yeckel and Berger, 1998). Pissadaki et al. (2010) investigated how timing and spatial variations in synaptic inputs to the distal and proximal dendritic layers of a CA1 pyramidal cell model influenced the cell's firing pattern. Introduction of a temporal delay in the activation of the two layers acted as a switch between excitability modes: short delays induced bursting, while long delays caused low frequency RS. Such activity-induced changes in neuronal firing patterns suggest a key role of these patterns in information processing in the brain.

Apart from ionic and synaptic effects, dendritic morphology has also been suggested to influence the firing behavior of neocortical and hippocampal pyramidal neurons (Bilkey and Schwartzkroin, 1990; Chagnac-Amitai et al., 1990; Mason and Larkman, 1990; Yang et al., 1996). Previous studies reported that IB neurons are characterized by large cell bodies, long and extensive apical dendritic trees, and axons that tend to ramify in subcortical and brainstem nuclei (Kelly and Wong, 1981; Games and Winer, 1988; Ojima et al., 1992). RS neurons on the other hand have smaller cell bodies, smaller dendritic arborizations with fewer oblique branches that end without terminal tufts, and their axons project via callosal connections to sensory cortices in the other hemisphere and to corticostriatal projections (Games and Winer, 1988; Rüttgers et al., 1990; Ojima et al., 1992; Winer and Prieto, 2001; Hattox and Nelson, 2007). Such differences in anatomical features have been associated to the different firing patterns in previous computational modeling studies (Mainen and Sejnowski, 1996; Krichmar et al., 2002; Van Ooyen et al., 2002). However, these earlier studies were qualitative and limited in the sense that they focused exclusively on the apical trees of morphologically very distinct cell classes (Mainen and Sejnowski, 1996; Krichmar et al., 2002).

Here, we use a modeling approach to investigate in a quantitative manner the effects of neuronal morphology (dendritic size and dendritic topology) on the firing behavior of 112 three-dimensional reconstructions of layer V PFC pyramidal cells. By systematically varying the basal and/or apical dendritic trees of these neurons as well as the distribution of ionic mechanisms along their dendritic trees, we predict which of the morphological parameters (diameter, total length, volume, branch number) and/or passive properties (Mean Electrotonic Path length—MEP) can best discriminate between IB and RS neurons. Considering that dendritic morphology alterations may lead to many pathological conditions, such as Alzheimer's disease and epilepsy (Yamada et al., 1988; Moolman et al., 2004; Teskey et al., 2006), the results of our modeling study are instrumental in uncovering, in a systematic way, the underlying mechanisms by which dendritic morphology and its alterations affect neuronal firing behavior.

## Materials and methods

### Morphological data

**Figure 1:**
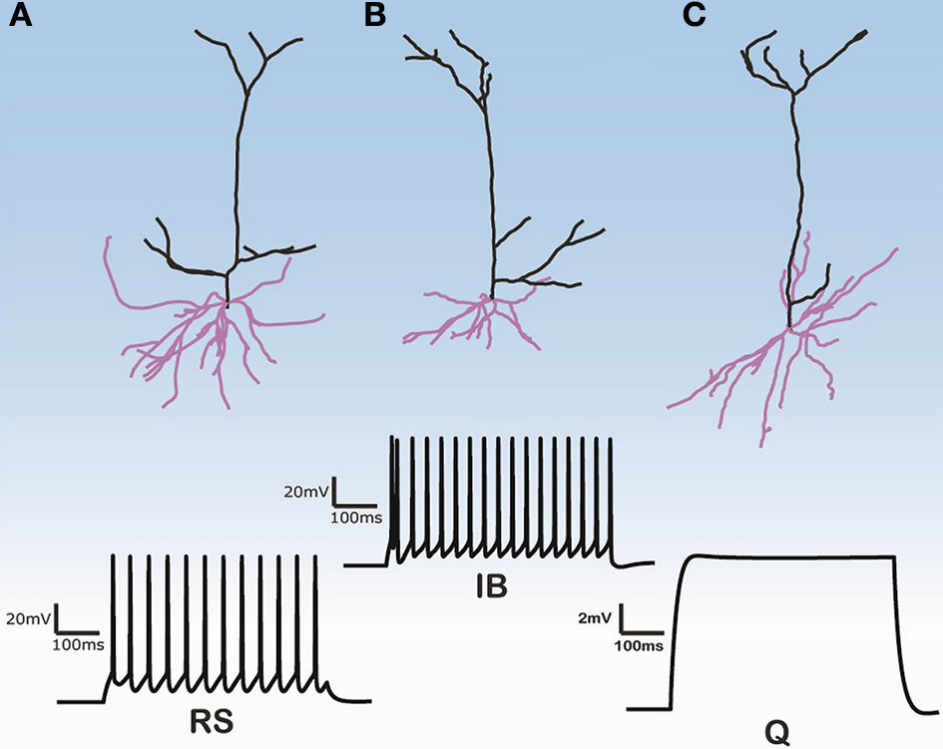
**Morphologies of three model cells and their responses to a somatic depolarization of 0.35 nA multiplied by the normalization factor for the input resistance**. Upper panel: The somatic compartment is the same in all model cells but apical and dendritic trees are taken from three distinct reconstructed morphologies. Lower panel: Cells either display regular spiking with no adaptation **(A)**, are intrinsic bursting **(B)**, that is an initial burst (1–2 spikes) followed by a regular spiking or are silent **(C)**.

Three-dimensional morphological data of 112 layer V pyramidal cells (PC) from the rat PFC were obtained from the NeuroMorpho database (http://neuromorpho.org) (see Supplementary Tables S1, S2 for morphological features of basal and apical dendritic trees of all 112 pyramidal cells). These neurons were previously reconstructed in the Smith lab from the brains of adult Long-Evans rats at 2–4 months of age (Bergstrom et al., 2008). No data regarding the size, shape and distribution of spines in the estimation of the dendritic surface area were provided. Images of three of these 112 cells are shown in Figure 1. The images of the remaining cells are available at the abovementioned web site.

The digitized cell reconstructions were acquired in SWC format (Ascoli, 1999). All SWC files were checked for any morphological reconstruction inconsistencies before being converted into the HOC format and loaded into the NEURON neural simulator (Hines and Carnevale, 1997).

### Pyramidal cell model

For all model cells, unless mentioned otherwise, we assumed a uniform membrane resistance of R_m_ = 30 kΩ cm^2^ in the soma and the axon. In the basal dendrites, the membrane resistance decreased sigmoidally up to half of the somatic value, according to the function ${\text{R}}_{\text{m}}(\text{x})=30-\frac{15}{1+e\frac{10-x}{5}}$. Similarly, the membrane resistance in the apical dendrites decreased sigmoidally up to half of the somatic value according to the formula ${\text{R}}_{\text{m}}(\text{x})=30-\frac{15}{1+e\frac{300-x}{50}}$ (Stuart and Spruston, 1998). A uniform intracellular resistivity R_a_ = 210Ω cm and a specific membrane capacitance (C_m_) of 1.2 μF cm^−2^ were used in the soma, axon, apical and basal dendrites. The resting membrane potential was set at −66 mV.

Active mechanisms included two types of Hodgkin–Huxley-type Na^+^ currents (transient: I_Naf_; persistent I_Nap_;), three voltage-dependent K^+^ currents (I_Kdr_; I_A_; I_D_), a fast Ca^2+^ and voltage-dependent K^+^ current, I_fAHP_; a slow Ca^2+^-dependent K^+^ current, I_sAHP_; a hyperpolarization-activated non-specific cation current (I_h_); a low-voltage activated calcium current I_CaT_ and three types of Ca^2+^- and voltage-dependent calcium currents (I_caN_; I_caR_; I_caL_). In all cells, the conductance of I_Naf_ was highest in the axon, and increased in the soma and apical dendrites compared to basal dendrites (González-Burgos and Barrionuevo, 2001). The conductance of all three different K^+^ currents was decreased in the apical dendrites compared to the soma (Korngreen and Sakmann, 2000; Schaefer et al., 2007). Both fast and slow AHP currents were present in the soma and much less in the apical dendrites (Lorenzon and Foehring, 1992). The h-current conductance increased sigmoidally, in the apical tree, reaching a maximum value that was10 times greater than the somatic value (Day et al., 2005; Kole et al., 2006). This increase was not implemented in the basal dendrites (Nevian et al., 2007). The mathematical formalism of the pyramidal neuron model used in this study was based on the model of Sidiropoulou and Poirazi (2012) and can be found in the Supporting Online Material (SOM). The parameter values of all active mechanisms are reported in Supplementary Table S3 in the SOM.

### Simulations setup

Cell “C3_5” (as referred in the NeuroMorpho database) was selected as the control morphology because it was previously used and extensively validated with respect to passive and active membrane properties as well as apical and basal dendritic responses against experimental data by Sidiropoulou and Poirazi (2012) (see Supplementary Figure S1 in Sidiropoulou and Poirazi, 2012 study). To investigate the effects of dendritic tree variability on firing behavior, we kept the soma of cell “C3_5” and varied the apical and basal trees attached to this soma using the 112 PFC layer V PCs previously described in Section “Morphological Data.” Which apical and/or basal tree(s) were attached depended on the specific experiment (see “Results” section for details). The input resistances of all simulated cells were calculated by measuring the steady-state voltage change in response to a 500 ms hyperpolarizing current pulse (0.1 nA). Previous studies (Washington et al., 2000; Krichmar et al., 2002) have shown that dendritic morphology has a strong effect on input resistance. In order to ensure equivalent depolarization in each model cell and eliminate the influence of the input resistance on the electrophysiological response to current injections, the current injections were scaled by the ratio of the cell's input resistance to that of the control cell (cell “C3_5”) multiplied by a constant factor of 0.35 (i.e., the current 0.35 nA), where cell “C3_5” first spiked). Thus, simulations consisted of depolarizing the PFC pyramidal cell model's soma with a normalized injection current, such that the initial instantaneous depolarization was equivalent for all cells, and recording the membrane potential at the soma.

### Morphological parameters

Morphological parameters were obtained directly from the three-dimensional neuroanatomical description and included the dendritic size (median diameter, total length, volume, and branch number). The MEP for each apical and basal dendritic tree was also derived. To estimate the median diameter, we first calculated the diameter of each cylindrical section of the dendritic tree (basal or apical) of each cell (112 cells in total) and then took the median value. To estimate the total length we summed the length of all sections of each tree (basal or apical) for all cells. To estimate the branch number, we calculated the branch number of each tree (basal or apical) for all cells.

The volume was estimated using the following equation:

(1) $$V=\sum_{i=1}^{N}\pi \;\cdot \;L_{i}\;\cdot \;{\left(D_{i}/2\right)}^{2}$$

where for every cylindrical section *i* of each tree, *L*_*i*_ is the length and *D*_*i*_ is the diameter. We estimated MEP as in Van Elburg and van Ooyen (2010). More specifically, to obtain the MEP of a dendritic tree, we first normalized the length *l*_*i*_ of each terminal, intermediate or root section *i* with respect to its electrotonic length constants λ_*i*_, yielding a dimensionless electrotonic length Λ_*i*_ = *l*_*i*_/λ_*i*_, in which λ_*i*_ was defined as:

(2) $$\lambda_{i}=\sqrt{\frac{b_{i}\;\cdot \;r_{m}}{2\;\cdot \;r_{a}}}$$

where *b*_*i*_ was the radius of dendritic section *i*, and *r*_*m*_ and *r*_*a*_ were constants denoting the specific membrane resistance and the intracellular resistivity, respectively. The MEP of a dendritic tree with *N* terminal sections was then given by:

(3) $$MEP=\frac{1}{N}\sum_{j=1}^{N}P_{j}$$

where *P*_*j*_ was the sum of the electrotonic lengths Λ_*i*_ of all dendritic sections in the path from the tip of terminal section *j* to the soma.

### Data analysis

We used two types of active mechanism distributions in the basal dendritic trees of cells: (1) uniform (i.e., the conductance of an active mechanism does not depend on the distance from the soma), and (2) non-uniform (distance dependent) (see Table S3). For each distribution of active mechanisms, the firing behaviors of the model cells were classified into two categories: (1) RS, and (2) IB. A model cell was considered as IB if the interspike interval (ISI) of the first two spikes in its spike train was smaller than 20 ms. Otherwise, the cell was considered as RS. For each firing behavior category (RS or IB), we estimated the median diameter, volume, MEP, branch number, and the total length of the basal or apical tree. A non-parametric statistical Mann–Whitney *U*-test was performed to test if these features were significantly different between the two categories.

In addition to detecting statistical differences, features such as the diameter, total length, MEP, volume, or branch number were used to classify each model cell as an RS or an IB via the use of a probabilistic Bayesian classifier. Probability distributions for the two categories (“training”) were estimated using 80% of the total number of cells (N_RS_ + N_IB_) and the classifier's classification accuracy was measured on the remaining 20% of cells (“testing”). This procedure was repeated 10 times, using different randomly selected training/test sets of the same size. The performance accuracy reported here was evaluated solely on the 10 test sets, namely the 20% of the samples that were not used for training. The performance reported is the average over the 10 different trials. If the sizes of the RS (N_RS_) and IB (N_IB_) samples were unequal (e.g., N_RS_ > N_IB_), then we set the size of the larger sample equal to the size of the smaller one, and used the 80% of each sample for training and the remaining 20% for testing. Classification performance was assessed using the sensitivity, specificity and accuracy metrics. Given a two class problem with Negative and Positive examples, sensitivity refers to the percentage of true Positives that are correctly identified, whereas specificity refers to the percentage of true Negatives that are correctly identified as such. Accuracy measures the proportion of true Positives and true Negatives correctly identified. The larger the sensitivity, specificity, and accuracy values, the better the classification.

### Implementation

The simulations were run in the NEURON simulation environment (Hines and Carnevale, 1997) and simulations were executed on a parallel cluster (8 core Intel Xeon processors). Data analysis was performed using MATLAB (The MathWorks Inc., Natick, Massachusetts) and was adapted from the Cuntz et al. (2010) work. The source code of the model is available upon request to the corresponding author at poirazi@imbb.forth.gr. The source code of the original model by Sidiropoulou and Poirazi (2012) can be found on ModelDB (accession number: 144089).

## Results

The firing behavior of the 112 simulated PFC layer V pyramidal cells in response to simulated somatic depolarization varied greatly. Model cells were either quiescent, i.e., with no spike response, displayed RS with no adaptation, or IB, that is an initial burst followed by RS. Figure 1 shows the responses of the two cell categories investigated in this simulation study, namely RS and IB, plus the quiescent state (Q) seen in just a few model cells. Images of the reconstructed cellular morphologies are superimposed (Figure 1, upper panel). Note that spiking activity was measured in response to a somatic depolarization of 0.35 nA multiplied by the normalization factor of the input resistance (see Materials and Methods). Indicative voltage traces before this normalization are shown in Supplementary Figure S1. For small currents (0.2 nA) IB cells displayed a characteristic fast after-depolarization (fADP) that generated the burst profile for larger currents (0.35 nA, Supplementary Figure S1). Since the channel distribution, Ca^2+^ concentration, soma volume and depolarization levels were the same across all cells, the firing behavior variation can be attributed to differences in the apical and basal dendritic morphology of the neurons.

**Figure 2:**
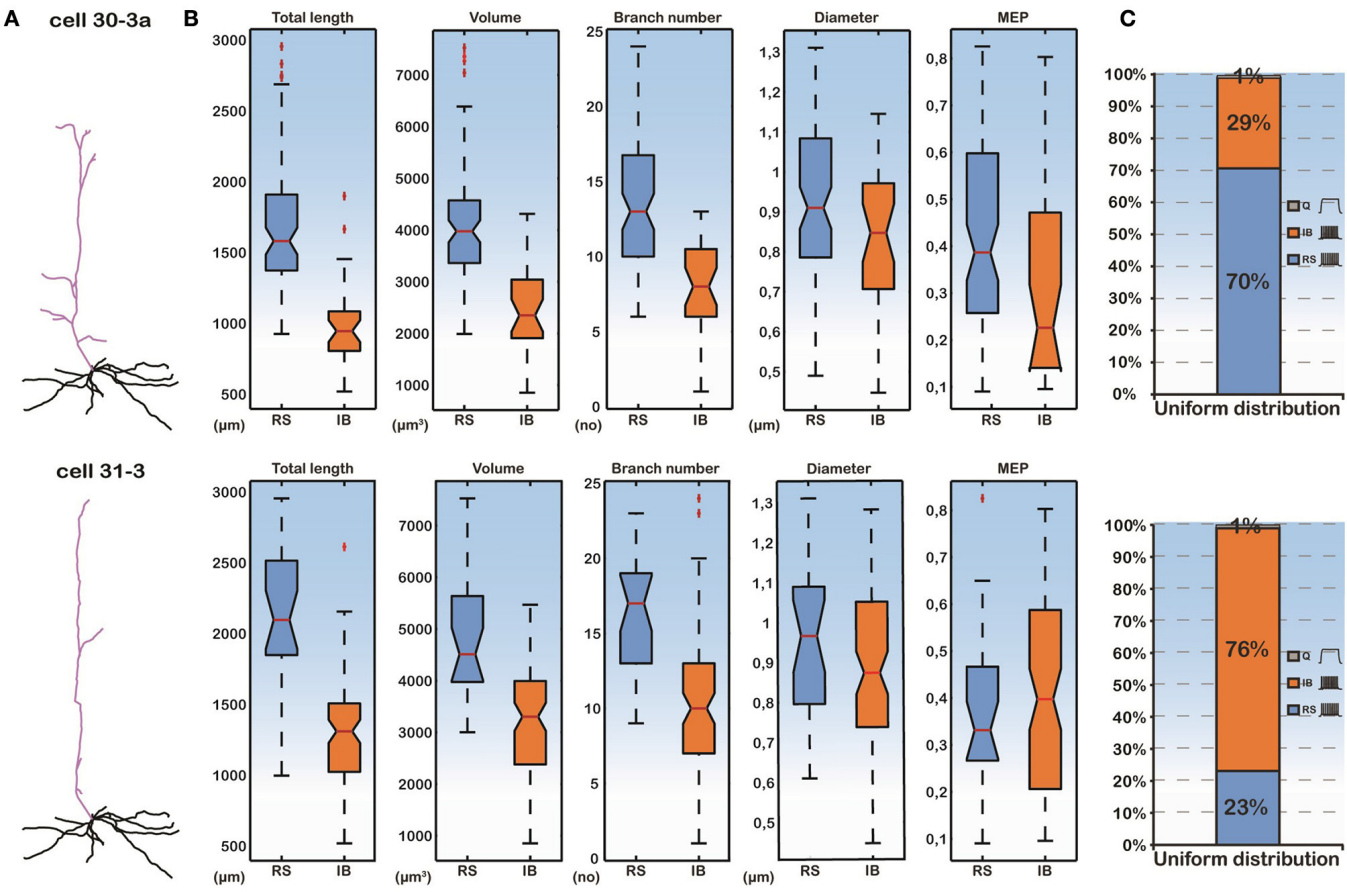
**(A)** Categorization of model cell firing patterns when using two different apical dendrites (a complex “30-3a” and a simple “31-3”) and 112 different basal dendrites. Ionic mechanisms are distributed uniformly along the basal tree. **(B)** Box plots of all morphological features of the basal trees and the MEP for the RS and IB categories. Differences in the features are statistically significant between the two categories. **(C)** Proportions of RS, IB, and Q cells are significantly different in the “30-3a” and “31-3” model cell cases.

To isolate the effect of basal dendritic morphology variations from those of the apical dendritic tree, on the firing behavior of layer V PFC pyramidal cells, we created two additional models. As before, the soma of both of these models corresponded to the soma of cell “C3_5” and their apical dendritic trees were taken from cells “30-3a” (hereby termed “complex apical model”) and “31-3” (hereby termed “simple apical model”) in our neuron pool (Figure 2A). These two apical trees were selected because they represented a simple and a complex morphology (Supplementary Table S1), in an effort to approximate the two extreme cases where the morphology of the apical tree may influence responses. The 112 different basal trees were subsequently attached to both of these models and the resulting somatic responses to the normalized somatic current injection were measured (Figure 2). We observed that in the complex apical model, the distribution of firing patterns in the 112 cells with different basal trees was 70% RS, 29% IB, and 1% quiescent (Figure 2C). In the simple apical model however, these proportions were reversed. We measured 23% RS, 76% IB, and 1% quiescent responses (Figure 2C). These results are in agreement with previous findings regarding the significant effect of the apical morphology on neuronal firing patterns (Chang and Luebke, 2007).

Importantly, while the apical tree morphology introduces a bias in neuronal output (seen in the different distributions of RS vs. IB model cells in Figure 2C), given the same apical dendrite (complex or simple) the different firing patterns observed are solely due to the different basal morphologies. As shown in Figure 2B, the total length, the volume and the branch number of the basal dendritic tree were statistically different (*p*-value < 0.001) between the RS and IB spiking profiles, irrespectively of the apical tree used. In fact these features could also be used to predict the model cell's firing pattern via a probabilistic classifier (see Supplementary Figure S2). This suggests that, apart from the morphology of the apical tree, basal tree morphology also contributes to the spiking profile of layer V PFC pyramidal neurons.

**Figure 3:**
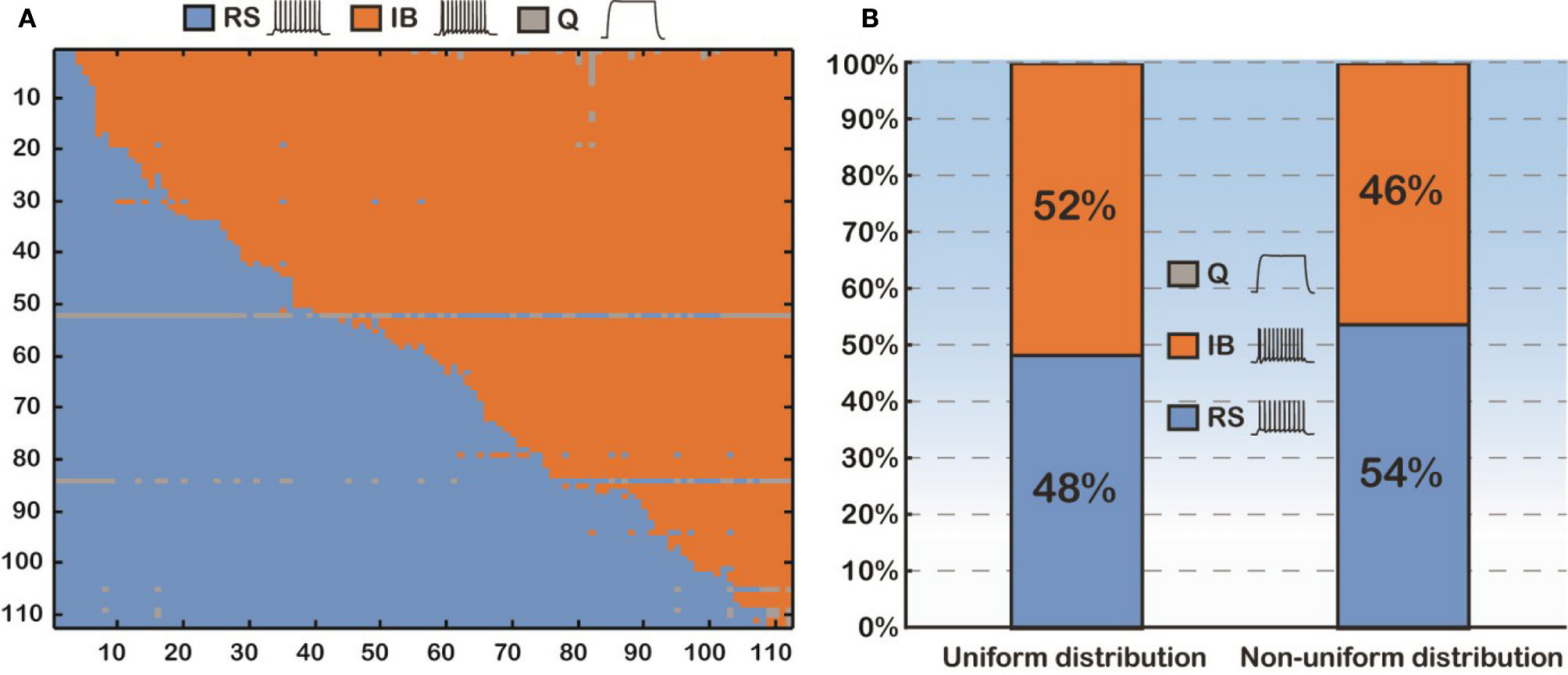
**(A)** Confusion matrix of 12544 (112 × 112) combinations of dendritic trees (basal and apical) sorted according to the firing pattern of the model cells. **(B)** Proportions of the model cells' firing behavior (RS vs. IB). Uniform distribution means that the conductance of an active mechanism is not dependent on the distance from the soma. Non-uniform distribution means the conductance of an active mechanism is distance-dependent.

Thus, we sought to thoroughly examine how basal dendritic tree variability may influence the firing behavior of layer V PFC pyramidal cells, irrespectively of the apical tree morphology. Toward this goal, we again used the soma of cell “C3_5,” but this time varied both its basal and apical dendritic trees, thus generating 112 × 112 morphological combinations (Figure 3A). For each of the 112 basal trees, 112 different apical trees were attached and somatic response patterns to normalized current injection were recorded as detailed previously. As shown in Figure 3A, utilization of different basal trees (y-axis) greatly influenced the percentage of RS vs. IB firing patterns produced for each apical tree attached (x-axis). To somehow average the effect of apical tree morphology on the firing pattern of a model cell with a given basal tree we applied the following approach: for each basal dendritic morphology (row in Figure 3A), if more than half of the 112 model cells produced a RS response, then this basal dendritic morphology was considered an RS (that is it has the “tendency” to produce an RS profile, irrespectively of the apical tree used). Otherwise, the basal dendritic morphology was considered an IB. Out of the 112 basal trees used, 54 (48%) were assigned an RS profile and 58 (52%) were assigned an IB profile (Figure 3B). Since in all these experiments the distribution of ionic mechanisms along the basal tree was uniform, we investigated the robustness of our findings in the presence of a non-uniform mechanism distribution (i.e., distance-dependent, see also Supplementary Table S3). The results were very similar: 46% of the basal trees were assigned an RS profile and 54% were considered IB (Figure 3B).

**Figure 4:**
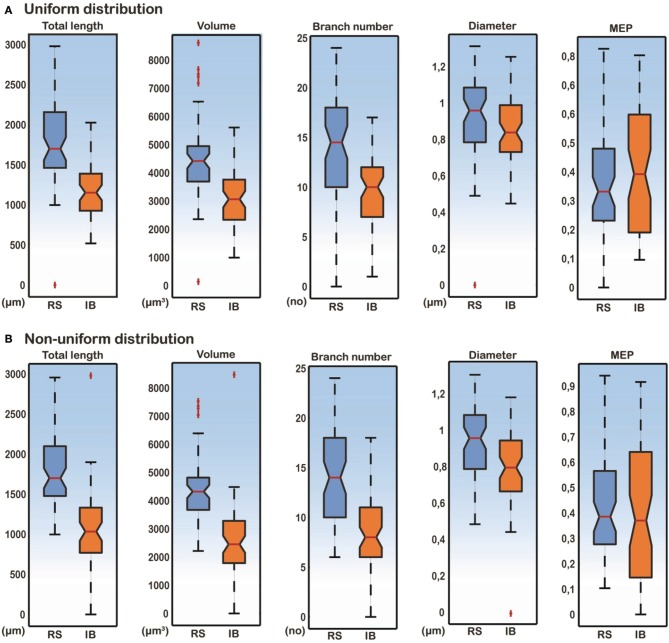
**Box-plots of total length, volume, branch number, median diameter, and MEP features for RS and IB model cells, under conditions of both uniform and non-uniform ionic mechanism distributions**. Total length, volume and branch number are the best discriminatory parameters of the RS and IB firing behavior for both uniform **(A)** and non-uniform **(B)** distributions.

A detailed analysis of the model cells that exhibited an RS or an IB profile showed that the basal trees of RS cells had a median diameter of 0.97 μm, a total length of 1739.2 μm, a volume of 4349.1 μm^3^, a MEP of 0.34 and a branch number of 15. The basal trees of IB model cells on the other hand, had a median diameter of 0.837 μm, a total length of 1152.3 μm, a volume of 2924.6 μm^3^, a MEP of 0.39 and a branch number of 10. A Mann–Whitney *U*-test showed a statistically significant difference (*p* < 0.01) for the total length, volume, and branch number between the RS and IB model cells, for both uniform and non-uniform distributions of the ionic mechanisms along the basal tree (see Table 1 for details). Box-plots of RS and IB median diameter, total length, MEP, volume, and branch number in the uniform and non-uniform distribution cases are depicted in Figure 4. It is clearly evident that in both cases the total length, volume and branch number are the parameters that differ the most between model cells exhibiting an RS vs. an IB firing behavior.

**Table 1:** **Median diameter, total length, MEP, volume, and branch number values for both RS and IB in the uniform and non-uniform cases**.

	**Diameter (μm)**	**Total length (μm)**	**MEP**	**Volume (μm^3^)**	**Branch number**
**UNIFORM**					
RS	0.967	1739.2	0.3391	4349.1	15
IB	0.837	1152.3	0.3921	2924.6	10
*p*-value	0.0061	3.37E-13	0.7532	1.50E-10	5.65E-09
**NON-UNIFORM**					
RS	0.963	1699.2	0.385	4322.3	14
IB	0.8375	1128.8	0.4528	2722.9	9
*p*-value	0.012	1.30E-12	0.9117	7.13E-12	3.71E-08

*A Mann–Whitney U-test showed a statistically significant difference (p < 0.01, alpha level = 0.05) for the total length, volume, and branch number between the RS and IB cells regardless of the distribution of ionic mechanisms*.

**Figure 5:**
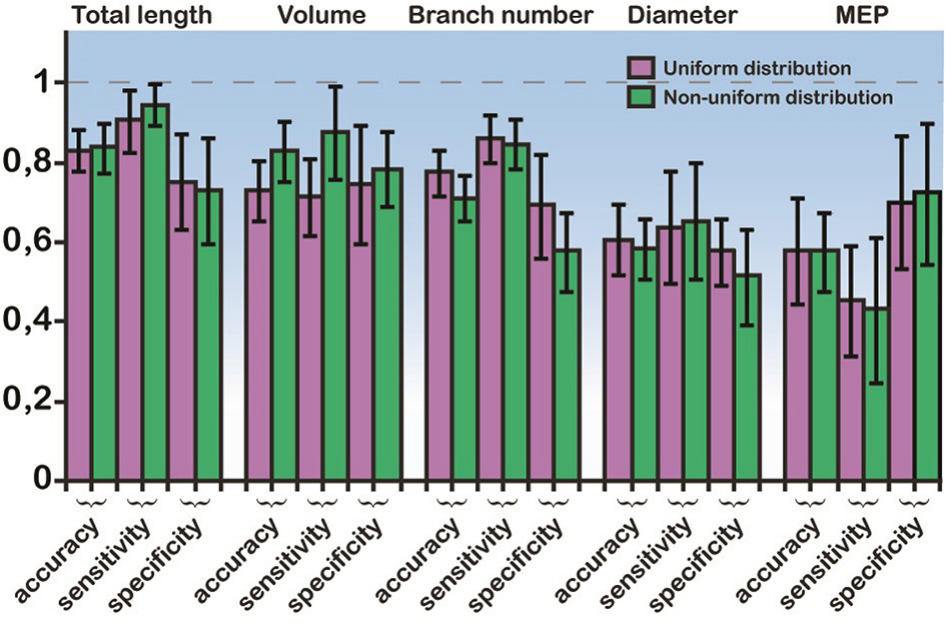
**Bayes classifier performance**. Sensitivity, specificity, and accuracy values for the classification when using a single feature: the mean diameter, the total length, the MEP, the volume, and the branch number of basal trees. Performance is shown for both uniform and non-uniform ionic mechanism distributions. The error bars are also depicted (black lines). Total length, volume, and branch number are the best discriminatory features for predicting whether a model cell will express an RS or an IB firing behavior.

In light of these statistical differences in anatomical features of RS vs. IB model cells, we next questioned whether the morphology of the basal tree has a determinant role in shaping the resulting response patterns, irrespectively of the apical tree. Toward this goal, we used a probabilistic classifier. Specifically, we employed a Bayes classifier (see Materials and Methods) and used the diameter, total length, MEP, volume, or branch number of basal trees to test whether these features can correctly predict the firing pattern (RS or IB) of the respective model cell. Prediction accuracy was evaluated using the sensitivity, specificity, and classification accuracy metrics. We found that the best discriminatory parameters (i.e., the parameters with the highest sensitivity, specificity, and accuracy values) were the total length, volume and branch number, for both uniform and non-uniform distributions of ionic mechanism (Figure 5). Given that the accuracy achieved when using, for example, the total length of the basal tree is very high (>80%), these results suggest that anatomical features of the basal tree determine to a very large extent the firing pattern of the resulting model cell.

**Figure 6:**
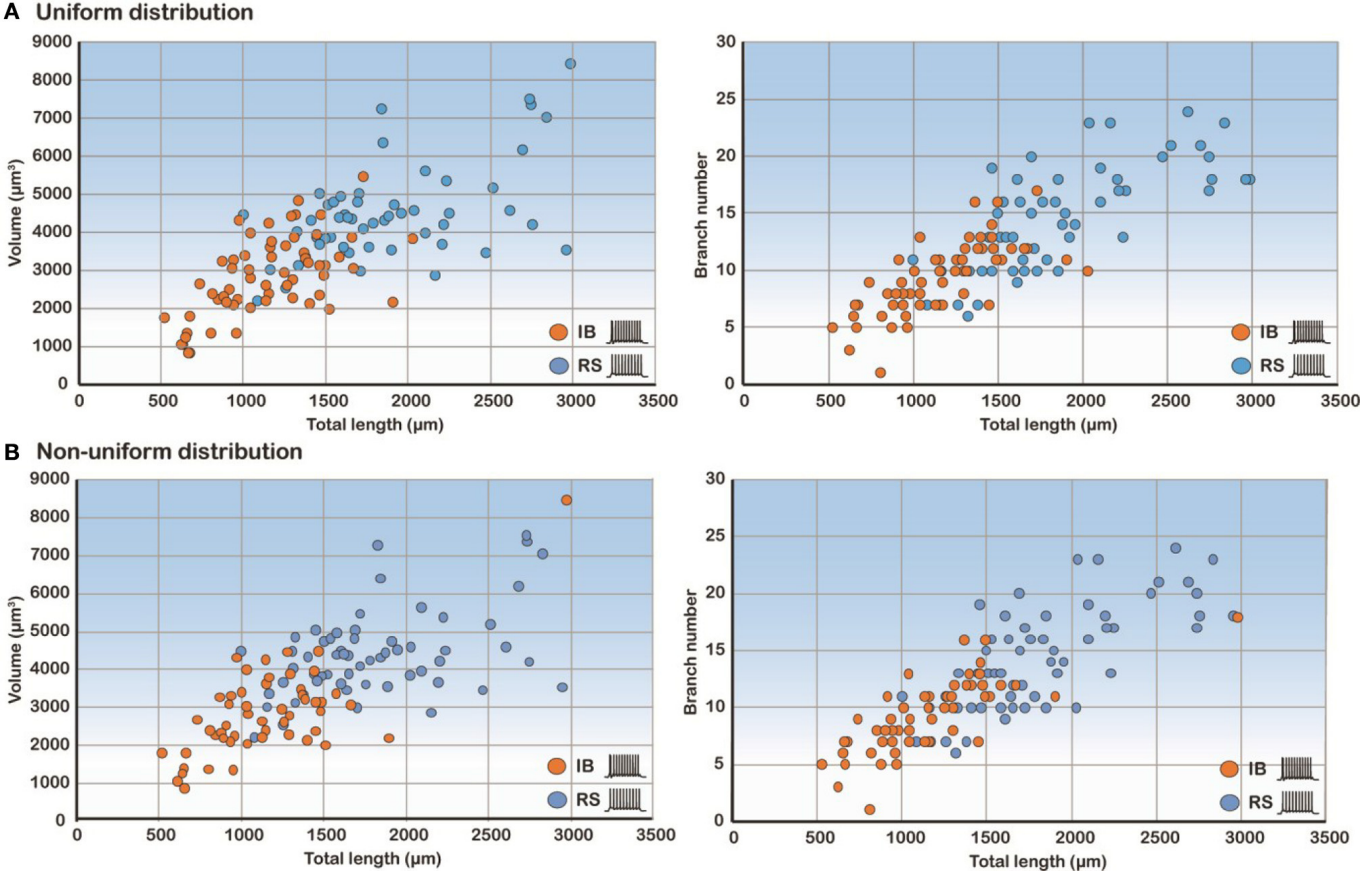
**Correlation analysis of the three most predictive features**. Scatter plots of volume and branch number as a function of the total length in the uniform **(A)** and non-uniform **(B)** distribution cases show that these features are highly correlated.

Since total length, volume and branch number of the basal tree were the morphological parameters that determined the electrophysiological profile, we also performed correlation analysis of these features. As expected, the volume and branch number correlate strongly with the total length in both uniform and non-uniform cases (Figure 6). These results explain why these three features have a high discriminatory power: any of them is sufficient to predict with high confidence the firing pattern of a model cell with a particular basal tree.

## Discussion

### General issues

A biophysically realistic model of a layer V PFC pyramidal cell was extended to quantitatively investigate the effects of dendritic morphology and distribution of ionic mechanisms (uniform vs. non-uniform) along its basal dendrites on its firing behavior. The model cell was extensively validated in a previous study (Sidiropoulou and Poirazi, 2012) from our group against a wealth of experimental data (Haj-Dahmane and Andrade, 1998, 1999; Fowler et al., 2007; Milojkovic et al., 2007; Nevian et al., 2007; Wang et al., 2008; Sidiropoulou et al., 2009) casting it as a faithful representation of a biologically realistic layer V PFC pyramidal cell. Using this experimentally validated model we systematically varied its basal and apical dendritic trees as well as its ionic mechanism distribution along the basal dendritic trees to parametrically investigate which morphological parameters (diameter, total length, volume, and basal number) and passive properties (MEP) discriminated the cell's firing behavior as an RS or an IB when it was stimulated with a somatic current injection. Our simulation study has quantitatively showed that total length, volume and branch number of the basal dendritic tree are the best morphological parameters to discriminate the cell as an RS or an IB regardless of what is the distribution of ionic mechanisms along the basal trees and irrespectively of the apical tree. Varying combinations of the basal and the apical dendritic tree plexuses and their ionic mechanism distribution produced different cell type percentages indicating that the morphology of apical and basal trees has a strong effect on firing behavior. It should be noted ionic conductance changes are also likely to have an effect on firing patterns. However, the goal of this work was to dissect the effect of morphology from that of biophysics, therefore we chose the use of a model whose ionic mechanisms have been extensively validated against experimental data, to ensure that our analysis is within realistic bounds for these mechanisms. Under such realistic conditions, this work shows in a quantitative manner that specific anatomical features of basal trees in layer V PFC pyramidal neurons are largely determinant of the cells' firing pattern.

### What have we learned from this model?

The major finding of our simulation study was that the morphological parameters that best discriminated our cells as RS and IB were the total length, volume, and branch number of the basal dendritic tree. This finding was independent of the distribution of ionic mechanisms (uniform vs. non-uniform) along the basal tree (see Table 1 and Figures 2, 4, 5), and insensitive to the use of specific apical trees (Figure 2 and Supplementary Figure S2). The total length of the basal dendritic tree of the RS cells was found to be significantly larger than the total length of the basal dendritic tree of the IB cells. That means that the basal dendritic trees of RS cells are more extensive than the basal trees of IB cells. This result is in line with recent experimental evidence (Chang and Luebke, 2007) and supported by our finding that the branch number in RS model cells is significantly larger than that of IB model cells (Chang and Luebke, 2007). Chang and Luebke (2007) have also reported that the total length and branch number of RS cells in layer V of PFC are greater than that of IB cells in the same layer. On the other hand, another experimental study has shown that IB cells in layer V of the somatosensory neocortex in rats have extensive basal dendritic trees, and their axons tend to ramify in infragranular layers, while RS cells in same layer have smaller dendritic arborizations and their axons ramify to supragranular layers (Chagnac-Amitai and Connors, 1989; Connors and Long, 2004). Yang et al. (1996) showed in layers V-VI of the PFC that RS' proximal, but not basal, dendritic trees bifurcated less profusely than those of IB cells. We think that these variations are region dependent. The basal total length and branch numbers in layer V basal trees of RS pyramidal cells are greater than those of layer V IB pyramidal cells only in the PFC (Chang and Luebke, 2007).

In addition, our simulation study showed that the volume of the basal dendritic trees of RS cells is significantly greater than the volume of IB cells (Figure 4). A larger volume would mean a greater attenuation of the current flowing through the basal tree making the cell less excitable. A larger value of the total length and branch number of the basal tree would also contribute significantly to the reduced cell excitability. In our study, the diameter and the MEP of the basal trees were not the best discriminatory parameters of the cell's firing behavior. This result is contrary to the one reported by Van Elburg and van Ooyen (2010), where MEP was the best discriminatory parameter of the cell's output. One potential explanation for this discrepancy is that dendritic tree size and topological structure was altered without changing the total dendritic length to induce RS or IB firing in that study.

The percentages of RS and IB cells in our simulation study varied greatly, depending on the morphology of the apical tree used and the distribution of ionic mechanisms. In the complex model cell case with a uniform mechanism distribution 70% of the cells were RS, 29% were IB and 1% were quiescent. Use of a simpler apical tree reversed these percentages, resulting in 23% RS vs. 76% IB cells (Figure 2). On average however, across all possible apical trees, the percentages of RS and IB cells were 52% and 48%, respectively, for a uniform mechanism distribution. Changing the distribution into a non-uniform only slightly affected these numbers: 46% IB vs. 54% RS cells. Experimental studies (Yang et al., 1996) have reported similar percentages of IB cells in the layer V PFC (64% IB). The percentages of RS cells though were significantly smaller than ours (19% RS cells in Yang et al., 1996). We think this is due to the fact that in our study we only had two classes of cells (RS and IB), whereas in theirs the cells were classified into four types [RS, IB, ROB, and IM (intermediate)]. Also, in the Yang et al. (1996) although the cells studied were from the prelimbic area of the rat PFC as in ours, nevertheless their sample was an under-representative one of PFC neurons projecting to nucleus accumbens (NAc). In fact almost 80% of their PFC → NAc cells were bursting (IB and ROB) ones.

### What is next?

Several extensions to the basic idea deserve further consideration. One such idea is to thoroughly investigate how dendritic morphology affects the somatic firing behavior of a layer V PFC pyramidal cell when the cell's dendritic trees are driven by excitatory synaptic inputs. For example, nonlinear dendritic integration of spatially segregated inputs or spatially clustered synapses may have a much larger impact on the firing behavior of our model cells. Cortical and subcortical excitatory inputs drive PFC pyramidal cell's apical, proximal and basal dendrites, respectively. Each input may convey different information to the pyramidal cell. An experimental study (Schwindt and Crill, 1999) investigated the mechanisms underlying burst and RS evoked by dendritic depolarization in layer V pyramidal neurons in the rat somatosensory cortex. They reported that small dendritic depolarizations evoked spikes consisting of repetitive bursts of action potentials. Larger dendritic depolarizations evoked regular spikes. Burst firing was due to the interplay of Na^+^ and Ca^2+^ spikes. Somatic depolarizations evoked only RS in almost all recorded cells. A recent simulation study investigated the impact of the cell's dendritic morphology on the ping-pong mechanism of burst firing reported earlier, under either somatic current injection or synaptic stimulation of the apical dendritic tree of a layer V pyramidal cell of a cat visual cortex (Van Elburg and van Ooyen, 2010). They reported that burst firing is heavily dependent on the branching structure of the tree. However, none of these studies investigated explicitly the effects of synaptic stimulation of the basal dendritic trees of layer V pyramidal cells on their firing behavior.

Another idea is to investigate the role of basal and apical dendritic inhibition on the firing behavior of PFC pyramidal cells with varying dendritic morphologies. Recent optogenetic studies in the hippocampus have shown that dendritic inhibition can modulate the pyramidal cell somatic output more efficiently than somatic inhibition (Lovett-Barron et al., 2012). Silencing of dendritic inhibition allows NMDA dendritic spikes to turn the PCs from regular spikers to bursters (Lovett-Barron et al., 2012). A recent experimentally based theoretical study (Gidon and Segev, 2012) offered new insights into how dendritic inhibition controls dendritic excitability and affects the firing behavior of a neuron. They showed that distal “off-path” rather than proximal “on-path” inhibition effectively dampens proximal excitable dendritic hotspots, as it operates as a global threshold mechanism that powerfully controls the neuron's output. Varying the morphological parameters (diameter, total length, volume, MEP, etc.) of the dendritic segments at which inhibition impinges onto may uncover a different role of inhibition, perhaps that of a local as opposed to a global threshold setter.

## Concluding remarks

In summary, our computational work provides quantitative evidence that there is strong correlation between the firing behavior of pyramidal cells in layer V of the PFC and their dendritic morphology variations. We predict that, under realistic conditions for ionic mechanisms, irrespectively of their distribution along the basal tree, the total length, volume and branch number of basal dendritic trees determine to a large extent whether the firing pattern of the cell will be an RS or an IB. When both basal and apical dendritic trees varied, then the percentages of cells in the two categories did not change. These findings are likely to have serious implications in the information processing capabilities of pyramidal cells in layer V of the PFC in both normal and pathological conditions.

## Author contributions

Maria Psarrou, Stefanos S. Stefanou, and Athanasia Papoutsi designed experiments, run the simulations, analyzed the data, prepared the figures and helped with writing the manuscript. Alexandra Tzilivaki performed some simulations. Vassilis Cutsuridis helped with writing the manuscript and advised on certain experiments. Panayiota Poirazi designed the experiments, wrote/edited the manuscript and supervised the work.

### Conflict of interest statement

The authors declare that the research was conducted in the absence of any commercial or financial relationships that could be construed as a potential conflict of interest.
