# Supplementary material for: A simulation study on the effects of dendritic morphology on layer V prefrontal pyramidal cell firing behavior
Source: Front Cell Neurosci. 2014 Sep 16;8:287. doi: 10.3389/fncel.2014.00287 (PMC4165233; doi:10.3389/fncel.2014.00287)
Supplement: Supplementary file 1 [file DataSheet1.PDF]

Supporting Online Material

Maria Psarrou[1,3,4‡], Stefanos S. Stefanou[1, 2,‡], Athanasia Papoutsi[1,], Alexandra Tzilivaki[1,2], Vassilis Cutsuridis[1] and Panayiota Poirazi[1,*]

[1]Institute of Molecular Biology and Biotechnology, Foundation for Research and Technology – Hellas, Heraklion, Crete, Greece

[2]Department of Biology, University of Crete, Heraklion, Crete, Greece

[3]School of Computer Science, University of Hertfordshire , Hatfield, Hertfordshire, United Kingdom

[4]Science and Technology Research Institute, Hertfordshire, Hatfield, Hertfordshire, United Kingdom

‡ These authors contributed equally to the work

[*]*Corresponding author*: Panayiota Poirazi, Institute of Molecular Biology and Biotechnology (IMBB), Foundation of Research and Technology-Hellas (FORTH), N. Plastira 100, Vassilika Vouton, P.O.Box 1385, GR 700 13, Heraklion, Crete, Greece. E-mail: poirazi@imbb.forth.gr

SOM contains:

- Mathematical formalism
- Supplementary figure S1-S2
- Supplementary tables S1-S3

Mathematical formalism

The mathematical formalism of the layer V PFC pyramidal neuron model was based on the model of Sidiropoulou and Poirazi (2012). The parameter values of some ionic and synaptic currents were changed from the study of Sidiropoulou and Poirazi (2012) (see Supplementary Table S3 for details). In order to assist the readers of the paper, we list below the mathematical equations and parameter values (new and old) of the neuron model.

Distributions of biophysical mechanisms in the model neurons

All the biophysical mechanisms were differentially distributed along the somatodendritic axis, based on available experimental data.

a) Hodgkin–Huxley-type Na^+ currents (transient: I_{naf} persistent: I_{Nap})

The conductance of I_{Naf} was highest in the axon, and increased in the soma and proximal dendrites compared to distal and basal dendrite (Gonzalez-Burgos and Barrionuevo, 2001).

b) three voltage-dependent K^+ currents (I_{Kdr}; I_A; I_D).

The conductance of all three different K^+ currents was decreased in the apical dendrites compared to the soma (Korngreen and Sakmann, 2000; Schaefer et al., 2007)

c) a fast Ca^{++} and voltage-dependent K^+ current, I_{fAHP}; a slow Ca^{++}-dependent K^+ current, I_{sAHP} (Lorenzon and Foehring, 1992), which was present in the soma and much less in the apical dendrites

d) a hyperpolarization-activated non-specific cation current (I_h), whose conductance was increased, compared to the soma, sigmoidally with a maximum value of 10x that in the soma (Day et

al., 2005; Kole et al., 2006) but not the basal dendrites (Nevian et al., 2007)

e) a low-voltage activated calcium current I_{caT} (de la and Geijo-Barrientos, 1996)

f) four types of Ca^{++}- and voltage-dependent calcium currents (I_{caN}; I_{caR}; I_{caL}) (Lorenzon and Foehring, 1995)

Equations for all biophysical mechanisms used

The leak current

$I_L = g_L \cdot (V - E_L)$ $I_l = g_l * (V - E_l)$, where E_l = -65 mV

(1)

The fast sodium channel

$I_{Naf} = g_{Naf} \cdot (V - E_{Na})$ $I_{Naf} = g_{Na(fast)} * (V - E_{Na})$, where E_{Na}=55 mV

(2)

$$g_{Naf} = g \cdot m^2 \cdot h$$

$$\frac{dm}{dt} = \frac{m_\infty - m}{t_m}$$

$$m_\infty = \frac{ma}{ma + mb}$$

$$ma = \frac{-0.2816 * (v + 28)}{-1 + e^{\frac{-v+28}{9.3}}}$$

$$mb = \frac{0.2464 * (v+1)}{-1 + e^{\frac{v+1}{6}}}$$

$$t_m = \frac{1}{ma + mb}$$

$$\frac{dh}{dt} = \frac{h_\infty - h}{t_h}$$

$$h_\infty = \frac{ha}{ha + hb}$$

$$ha = \frac{0.0.98}{e^{\frac{v+23.1}{20}}}$$

$$hb = \frac{1.4}{1 + e^{\frac{-(v+25.1)}{10}}}$$

$$t_h = \frac{1}{ha + hb}$$

The persistent sodium channel (I_{Nap})

$I_{NaP} = g_{NaP} * (V - E_{Na})$, where E_{Na}= 55 mV (3)

$$g_{NaP} = g * m * h$$

$$\frac{dm}{dt} = \frac{m_\infty - m}{t_m}$$

$$m_\infty = \frac{ma}{ma + mb}$$

$$t_m = \frac{1}{ma + mb}$$

$$ma = \frac{-0.2816 * (v + 12)}{-1 + e^{\frac{-(v+12)}{9.3}}}$$

$$mb = \frac{0.2464 * (v - 15)}{-1 + e^{\frac{v-15}{6}}}$$

$$\frac{d\boldsymbol{h}}{dt} = \frac{\boldsymbol{h}_\infty - \boldsymbol{h}}{t_{\boldsymbol{h}}}$$

$$\boldsymbol{h}_\infty = \frac{\boldsymbol{h}a}{\boldsymbol{h}a + \boldsymbol{h}b}$$

$$t_{\boldsymbol{h}} = \frac{1}{\boldsymbol{h}a + \boldsymbol{h}b}$$

$$\boldsymbol{h}a = 2.8 * 10^{-5} * e^{\frac{-(v+42.8477)}{4.0248}}$$

$$\boldsymbol{h}b = \frac{0.02}{1 + e^{\frac{-(v-413.9284)}{148.2589}}}$$

The delayed rectifier potassium current (I_{Kdr})

$I_{Kdr} = g_{Kdr} * (V - E_K)$, where E_K= -85mV (4)

$$g_{Kdr} = g * n^4$$

$$\frac{dn}{dt} = \frac{n_\infty - n}{t}$$

$$n_\infty = \frac{na}{na + nb}$$

$$t = \frac{1}{na + nb}$$

$$na = \frac{-0.0.18 * (v - 13)}{-1 + e^{\frac{-(v+13)}{25}}}$$

$$nb = \frac{0.00544 * (v - 23)}{-1 + e^{\frac{(v-23)}{12}}}$$

The fast inactivating potassium current (I_A)

$I_A = g_A * n * l * (V - E_K)$, where E_K = -85 mV (5)

$$\frac{dn}{dt} = \frac{n_\infty - n}{tn}$$

$$\frac{dl}{dt} = \frac{l_\infty - l}{tl}$$

$$n_\infty = \frac{1}{1 + \alpha_n}$$

$$l_\infty = \frac{1}{1 + \alpha_l}$$

$$tn = \frac{betn}{qt * 0.1 * (1 + \alpha_n)}$$

$$betn = e^{\frac{0.001 * \left(-1.8 - \frac{1}{1+e^{\frac{(v+40)}{5}}}\right) * 0.39 * (v+1) * 9.648e4}{8.315 * (273.16 + celsius)}}$$

$$q_t = 5^{\frac{celsius - 24}{10}}$$

$$a_n = e^{\frac{0.001 * \left(-1.8 - \frac{1}{1+e^{\frac{(v+40)}{5}}}\right) * (v+1) * 9.648e4}{8.315 * (273.16 + celsius)}}$$

$$t_l = \frac{0.26}{v+50}$$

$$a_l = e^{\frac{0.001*3*(v+1)*9.648e4}{8.315*(273.16+celsius)}}$$

L-type calcium current (I_{CaL}) (McCormick and Huguenard, 1992)

$$I_{Ca(L)} = g_{CaL} * (m^2 *) * z^2 * \frac{v * F^2}{RT} * \frac{\left([Ca^{2+}]_i - [Ca^{2+}]_o * e^{\frac{-z*F*v}{R*T}}\right)}{1 - e^{\frac{-z*F*v}{R*T}}} \quad (6)$$

$$m = m + \left(1 - e^{-\frac{dt}{t_m}}\right) * (m_\infty - m)$$

$$t_m = \frac{3^{\frac{celsius-23.5}{10}} * 1.43}{(a+b)}$$

$$a = \frac{1.6}{1 + e^{-0.072*(v-15)}}$$

$$b = 0.02 * -\frac{v - 1.31}{1 - e^{\frac{v-1.31}{2.68}}}$$

$$m_\infty = \frac{1}{1 + e^{\frac{v+9}{-6}}}$$

N-type Calcium current (I_{CaN})

$$I_{Ca(N)} = g_{CaN} * m^2 * h * 0.\frac{025}{0.025 + [Ca^{2+}]_i} * (V - E_{Ca})$$, where E_{Ca}= 180mV (7)

$$\frac{dm}{dt} = \frac{m_\infty - m}{t_m}$$

$$m_\infty = \frac{1}{1+a}$$

$$a = e^{\frac{0.001*-3.4*(v+21)*9.648e4}{8.315*(273.16+celsius)}}$$

$$\frac{dh}{dt} = \frac{h_\infty - h}{t_h}$$

$$h_\infty = \frac{1}{1+b}$$

$$b = e^{\frac{0.001*2*(v+40)*9.648e4}{8.315*(273.16+celsius)}}$$

$$\frac{ds}{dt} = \frac{s_\infty - s}{t_s}$$

$$s_\infty = \frac{alpha}{1+alpha}$$

$$alpha = \left(\frac{[Ca^{+2}]_i}{b}\right)^2$$

where $t_m = 1.5$ ms and $t_h = 75$ ms.

R-type calcium current (I_{CaR})

$$I_{CaR} = g_{CaR} * m^3 * h * (V - E_{CaR}) \quad (8)$$

$$\frac{dm}{dt} = \frac{m_\infty - m}{t_m}$$

$$\frac{d\boldsymbol{h}}{dt} = \frac{\boldsymbol{h}_\infty - \boldsymbol{h}}{t_{\boldsymbol{h}}}$$

$$E_{CaR} = 1\frac{e^3 * R * (celsius + 273.15)}{2 * Faraday} * log\left(\frac{[Ca^{+2}]_o}{[Ca^{+2}]_i}\right)$$

$$m_\infty = \frac{1}{1 + e^{\frac{-(v+43.5)}{3}}}$$

$$\boldsymbol{h}_\infty = \frac{1}{1 + e^{v+50}}$$

where t_m = 8 ms and t_h = 1 ms.

Low threshold activated calcium current (I_{CaT})

$$I_{Ca(T)} = g_{CaT} * m^2 * h * (V - E_{Ca}) \quad (9)$$

$$\frac{dm}{dt} = \frac{m_\infty - m}{t_m}$$

$$m_\infty = \frac{1}{1 + a}$$

$$a = e^{\frac{0.001 * -3 * (v+36) * 9.648e4}{8.315 * (273.16 + celsius)}}$$

$$\frac{d\boldsymbol{h}}{dt} = \frac{\boldsymbol{h}_\infty - \boldsymbol{h}}{t_{\boldsymbol{h}}}$$

$$\boldsymbol{h}_\infty = \frac{1}{1 + b}$$

$$b = e^{\frac{0.001 * 5.2 * (v+68) * 9.648e4}{8.315 * (273.16 + celsius)}}$$

where t_m = 1.5 ms, t_h = 10 ms.

The fast calcium dependent potassium current (I_{fAHP})

$$I_{fAHP} = g * c^2 * (V - E_K) \tag{10}$$

$$\frac{dc}{dt} = \frac{(c_\infty - c)}{t_c}$$

$$c_\infty = \frac{ca}{c_a + c_b}$$

$$t_c = \frac{1}{c_a + c_b}$$

$$c_{\downarrow}a = ((-0.0064 * (v + 40 * \log_{\downarrow}10 \, [(1000 * [Ca^{\uparrow}(2+)]_{\downarrow}i) - 0.1152)))]) / e^{\uparrow}(-((v + 40 * \log_{\downarrow}10 \, [(1000 * [Ca^{\uparrow}(2+)]_{\downarrow}i) + 18]) / 12 - 1)$$

$$c_b = 1.7 * e^{\frac{-\left(v + 40 * \log_{10}[\left(1000 * [Ca^{2+}]_i\right) + 152\right)]}{30}}$$

The slow calcium dependent potassium channel I_{sAHP}

$$I_{sAHP} = g * m^2 * (V - E_K) \qquad \textbf{(11)}$$

$$\frac{dm}{dt} = \frac{m_\infty - m}{t_m}$$

$$m_\infty = \frac{a}{a + 1}$$

$$a = \frac{[Ca^{+2}]_i}{0.8}$$

$$t_m = 150 + \frac{0.8}{[Ca^{+2}]_i + 0.8}$$

where $[Ca^{+2}]_i$ is the internal calcium concentration (mM).

Hyperpolarization Activated Current (I_h)

$I_h = g_h * n * (V - E_h)$, where E_h = -10mV (12)

$$\frac{dn}{dt} = \frac{n_\infty - n}{t_n}$$

$$n_\infty = 1 - \frac{1}{1 + e^{\frac{(-90-v)}{10}}}$$

Where, for v>-10mV t_n=1ms, else:

$$t_n = 2 * \left(\frac{1}{e^{\frac{(v+145)}{-17.5}} + e^{\frac{v+16.8}{16.5}}} + 10 \right)$$

Slow inactivating potassium current (I_D)

$$I_D = g_D * a * b * (V - E_K) \quad (13)$$

$$\frac{da}{dt} = \frac{a_\infty - a}{t_a}$$

$$\frac{db}{dt} = \frac{b_\infty - b}{t_b}$$

$$a_\infty = \frac{1}{1 + e^{\frac{(v+34)}{6.5}}}$$

$$b_\infty = \frac{1}{1 + e^{\frac{(v+65)}{6.6}}}$$

where t_a=10 ms, t_b=3400 ms.

Supplementary Figure 1. Voltage response traces of the two cells shown in Figure 1, in response to a 500ms step pulse of 0.2 nA (top) and 0.35 nA (bottom) amplitude. Categorization as RS or IB was based on the normalized current response (see “Data analysis” section for details).

Supplementary Figure 2: Sensitivity, specificity and accuracy values of the mean diameter, total length, MEP, volume and branch number in the uniform case from two L5-PCs with different apical dendritic trees (“30-3a” and “31-3” cells (see supplementary table S1)). The error bars are depicted with black lines. The proportion of RS, IB and Q cells are significantly different in the “30-3a” and “31-3” cell cases.

Table S1. ***Morphological characteristics of apical dendritic trees of 112 layer V pyramidal cells in PFC***

Filenames	*Median Diameter (μm)*	*Total Length (μm)*	*Volume (μm³)*	*MEP*	*Branch number*
'0-2.CNG'	1,520755	1016,946	2630,159	0,681935	6
'0-2a.CNG'	1,413763	830,6306	1974,332	0,506118	5
'0-2b.CNG'	1,718308	763,7497	1677,329	0,657175	4
'0-2c.CNG'	1,487295	1189,424	1776,871	0,646102	8
'30-3.CNG'	1,414995	865,5099	1828,881	0,441016	5
'30-3a.CNG'	1,688391	765,5251	2390,732	0,278749	9
'30-3b.CNG'	1,316889	685,8608	1628,402	0,338642	4
'31-3.CNG'	2,466	704,9524	2646,502	0,241861	3
'31-3a.CNG'	1,829851	702,8049	1714,364	0,376754	3
'31-4.CNG'	1,797577	993,8177	2563,684	0,42028	6
'32-3.CNG'	1,089887	653,1048	1538,135	0,514435	3
'32-3a.CNG'	2,338104	966,5203	2819,49	0,524505	3
'32-3b.CNG'	1,154645	786,2907	1375,755	0,570049	4
'33-3.CNG'	1,64604	800,8679	2793,084	0,360812	5
'34-4.CNG'	1,916009	415,0335	1706,187	0,329441	2
'34-4a.CNG'	1,551037	747,3744	2248,729	0,324869	3
'34-4b.CNG'	1,452753	864,0126	2041,828	0,363595	6
'35-2.CNG'	1,248706	1010,011	2107,343	0,372864	5
'35-3.CNG'	0,993067	777,1771	1329,735	0,307727	5
'35-3a.CNG'	1,763037	717,7854	2263,873	0,308046	6
'36-4.CNG'	1,413923	1382,589	2577,66	0,324182	11
'36-4a.CNG'	2,467956	500,1195	2392,426	0,269644	0
'36-4b.CNG'	1,51497	1144,055	1662,021	0,358882	6
'37-3.CNG'	1,906427	1463,506	3278,657	0,396119	10
'37-4.CNG'	1,522114	849,8651	2444,844	0,29433	7
'37-4a.CNG'	1,241222	1429,802	2443,073	0,471283	9
'38-11.CNG'	1,211431	869,0001	1359,128	0,293347	7
'38-12.CNG'	2,169729	734,5407	1706,83	0,314482	6
'39-4a.CNG'	1,393745	1689,786	2721,096	0,297503	8
'39-5.CNG'	1,491536	672,202	1556,126	0,192619	4
'40-4.CNG'	1,402963	1091,408	1766,868	0,268811	8
'40-4a.CNG'	1,60555	943,5671	1576,573	0,287278	7
'40-4b.CNG'	1,012255	1509,953	1358,928	0,327787	14
'41-3.CNG'	1,550883	1742,868	4239,366	0,314421	11
'41-4.CNG'	1,291217	758,3111	1468,717	0,230397	5
'41-4a.CNG'	1,517866	1275,147	4152,229	0,276237	12
'43-3.CNG'	1,944398	918,5364	3235,513	0,185219	6
'43-3a.CNG'	1,202359	1268,713	2147,63	0,219759	6
'43-3c.CNG'	1,944398	918,5364	3235,513	0,185219	6
'43-4.CNG'	1,741572	1163,243	2995,836	0,133367	6

Table S2. ***Morphological characteristics of basal dendritic trees of 112 layer V pyramidal cells in PFC.***

Filenames	*Median Diameter (μm)*	*Total Length (μm)*	*Volume (μm^3)*	*MEP*	*Branch number*	*MEP (Uniform Mechanisms Configuration)*
'0-2.CNG'	1,61705	1577,044	3357,766	0,813978	12	0,717153
'0-2a.CNG'	1,716431	1295,456	2777,92	0,766166	8	0,675184
'0-2b.CNG'	1,378083	1513,556	1990,014	0,69601	11	0,608244
'0-2c.CNG'	1,663607	1468,687	4483,516	0,744624	12	0,653021
'30-3.CNG'	1,530686	844,5994	2253,323	0,581564	8	0,511022
'30-3a.CNG'	1,312665	1488,362	3125,117	0,677269	16	0,597853
'30-3b.CNG'	2,176563	973,905	4310,558	0,486523	8	0,431639
'31-3.CNG'	1,737754	1504,946	4744,518	0,511226	13	0,448877
'31-3a.CNG'	1,564395	2514,267	5199,309	0,535817	21	0,471863
'31-4.CNG'	1,603089	1260,855	2616,36	0,619983	11	0,544809
'32-3.CNG'	1,589702	1455,662	3139,664	0,514348	14	0,459108
'32-3a.CNG'	1,568934	668,0581	1789,065	0,660654	7	0,584532
'32-3b.CNG'	1,395354	1292,354	2284,38	0,616646	10	0,541141
'33-3.CNG'	1,791892	1694,066	5043,964	0,577297	15	0,507312
'34-4.CNG'	1,485252	2752,318	4206,393	0,371341	18	0,325297
'34-4a.CNG'	1,916041	2832,412	7041,633	0,354559	23	0,310576
'34-4b.CNG'	1,978084	1040,157	2819,584	0,480326	9	0,431893
'35-2.CNG'	1,534418	1688,399	4825,549	0,421799	20	0,371384
'35-3.CNG'	1,716064	1525,833	3870,845	0,451826	16	0,403069
'35-3a.CNG'	1,506027	1362,817	3457,551	0,430242	16	0,3775
'36-4.CNG'	1,501767	1846,041	6387,301	0,533095	18	0,466773
'36-4a.CNG'	1,685705	1542,045	4807,701	0,476963	13	0,421362
'36-4b.CNG'	1,424551	1892,418	3551,634	0,379894	15	0,335366
'37-3.CNG'	2,084446	2229,065	5374,456	0,432447	13	0,38097
'37-4.CNG'	1,700243	1607,908	4480,685	0,386555	18	0,340865
'37-4a.CNG'	1,796592	1254,532	3666,236	0,390755	11	0,34427
'38-11.CNG'	1,96151	733,1288	2667,821	0,412634	9	0,368042
'38-12.CNG'	1,895652	1154,225	3619,616	0,45011	11	0,397609
'39-4a.CNG'	1,676483	1641,311	3463,957	0,290849	11	0,256813
'39-5.CNG'	1,775479	1604,602	3633,991	0,371546	9	0,327543
'40-4.CNG'	1,789054	1950,87	4526,595	0,254938	14	0,223865
'40-4a.CNG'	1,535429	2243,916	4500,363	0,424626	17	0,372734
'40-4b.CNG'	1,767364	1149,475	4246,046	0,455519	10	0,402748
'41-3.CNG'	1,655388	2687,402	6182,592	0,383445	21	0,337317
'41-4.CNG'	1,428084	2198,793	3678,533	0,303221	18	0,266725
'41-4a.CNG'	1,845621	1444,622	3878,722	0,251654	13	0,222358
'43-3.CNG'	1,571319	1491,812	3832,695	0,295274	15	0,259809
'43-3a.CNG'	1,668814	2977,576	8467,459	0,264888	18	0,230869
'43-3c.CNG'	1,571319	1491,812	3832,695	0,295274	15	0,259809
'43-4.CNG'	1,237797	2155,878	2868,15	0,231774	23	0,203079

Table S3. ***Passive and active mechanisms along the various sections of the model neuron (RS neuron).***

**Mechanisms**	**Soma**	**Axon**	**Basal Dendrites**	**Apical dendrites**
R_m (KΩcm^2)	30	30	$30-\frac{15}{1+e^{\frac{10-x}{5}}}$ (non-uniform)<br><br>2*10^{-4} (uniform)	$30-\frac{15}{1+e^{\frac{300-x}{50}}}$ (non-uniform)
R_a (Ω cm)	210	210	210	210
C_m (µFcm^{-2})	1.2	1.2	1.2	1.2
g_{Naf} (S/cm^2)	0.155	0.31	0.0031	0.0062
g_{Kdr} (S/cm^2)	0.045	0.045	0.00405	4.5*10^{-5}
g_{Nap} (S/cm^2)	10^{-6}	0	10^{-5}	If distance from the soma <200µm: 10^{-5}<br>If distance from the soma >200µm: $\frac{5\cdot 10^{-5}\cdot x}{200}$ $\frac{(5 * 10^{-5} * x)}{200}$
g_A (S/cm^2)	7.5*10^{-4}	0	If distance from the soma <50µm: 1.5*10^{-3}<br>If distance from the soma >50µm: 3.75*10^{-3}<br><br>2.65*10^{-3} (uniform)	If distance from the soma <100µm: 7.5*10^{-4}<br>If distance from the soma >100µm and <300µm: $\frac{2.25\cdot 10^{-2}}{x}$<br>$\frac{2.25 * 10^{-2}}{x}$<br>If distance from the soma >300µm: 7.5*10^{-5}
g_D (S/cm^2)	1.2*10^{-3}	0	0	If distance from the soma <100µm: 1.2*10^{-3}<br>If distance from the soma >100µm and <300µm: $\frac{3.6\cdot 10^{-2}}{x}$<br>$\frac{3.6 * 10^{-2}}{x}$<br>If distance from the soma >300µm: 1.2*10^{-4}
g_{CaN} (S/cm^2)	3e-5	0	If distance from the soma <50µm: 10^{-6}<br>If distance from the soma >50µm: 3*10^{-6}<br><br>1.95*10^{-6} (uniform)	If distance from the soma <200µm: 10^{-6}<br>If distance from the soma >200µm: $\frac{9.6\cdot 10^{-5}\cdot x}{200}$
g_{CaT} (S/cm^2)	10^{-5}	0	0	If distance from the soma <200µm: 10^{-5}<br>If distance from the soma >200µm: $\frac{10^{-5}\cdot x}{200}$
g_{CaR} (S/cm^2)	3*10^{-5}	0	0	If distance from the soma <200µm: 1.5*10^{-5}<br>If distance from the soma >200µm:

				$3 \cdot 10^{-5} \cdot x/200$
g_{CaL} (S/cm^2)	$4,5*10^{-5}$	0	0	If distance from the soma <200μm: $4.5*10^{-5}$ If distance from the soma >200μm: $\frac{3 \cdot 10^{-4}}{x}$
g_{sAHP} (S/cm^2)	$2.5*10^{-2}$	0	0	If distance from the soma <200μm: $2.5*10^{-3}$ If distance from the soma >200μm: $2.5*10^{-5}$
g_{fAHP} (S/cm^2)	$2.7*10^{-2}$	0	0	If distance from the soma <200μm: $1.35*10^{-3}$ If distance from the soma >200μm: $2.7*10^{-5}$
g_h (S/cm^2)	$1.872*10^{-5}$	0	$1.872*10^{-5}$	$1.872*10^{-5} + \frac{1.62 \cdot 10^{-4}}{1+e^{\frac{300-x}{50}}}$
Calcium diffusion model	Yes	No	Yes	Yes

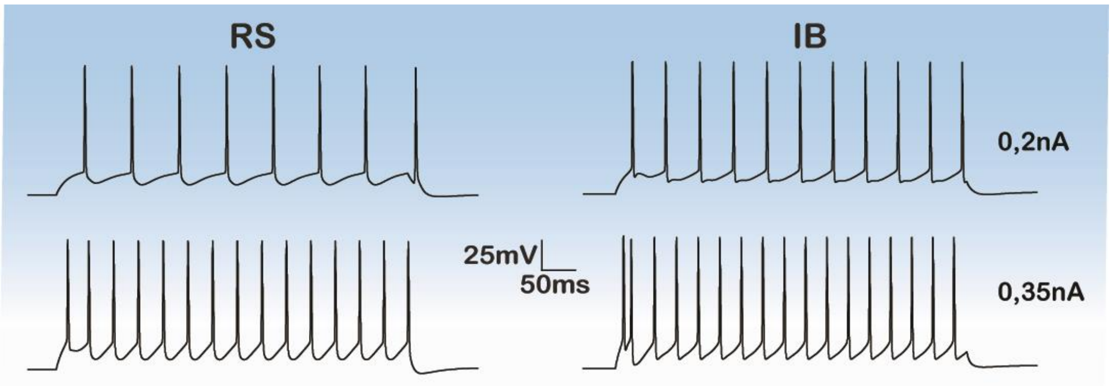

Supplementary Figure 1

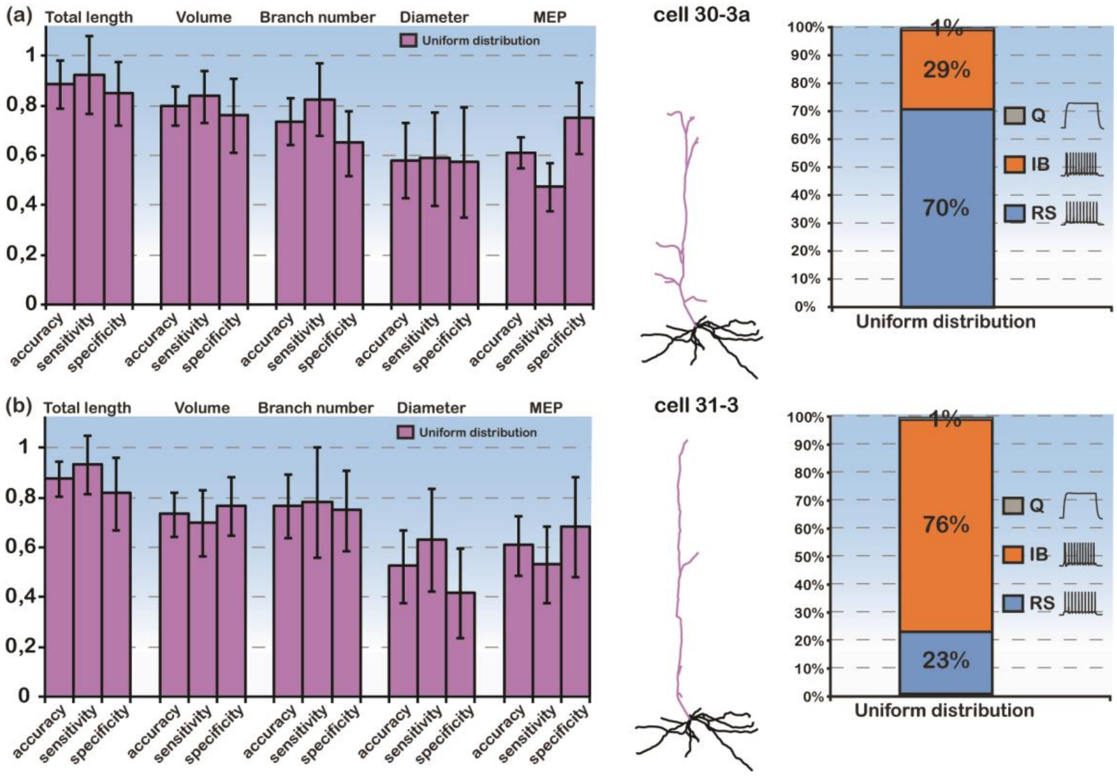

Supplementary Figure 2

References

Day M, Carr DB, Ulrich S, Ilijic E, Tkatch T, Surmeier DJ (2005) Dendritic excitability of mouse frontal cortex pyramidal neurons is shaped by the interaction among HCN, Kir2, and Kleak channels. JNeurosci 25:8776-8787.

de la PE, Geijo-Barrientos E (1996) Laminar localization, morphology, and physiological properties of pyramidal neurons that have the low-threshold calcium current in the guinea-pig medial frontal cortex. JNeurosci 16:5301-5311.

Gonzalez-Burgos G, Barrionuevo G (2001) Voltage-gated sodium channels shape subthreshold EPSPs in layer 5 pyramidal neurons from rat prefrontal cortex. J Neurophysiol 86:1671-1684.

Kole MH, Hallermann S, Stuart GJ (2006) Single Ih channels in pyramidal neuron dendrites: properties, distribution, and impact on action potential output. J Neurosci 26:1677-1687.

Korngreen A, Sakmann B (2000) Voltage-gated K+ channels in layer 5 neocortical pyramidal neurones from young rats: subtypes and gradients. J Physiol 525 Pt 3:621-639.

Lorenzon NM, Foehring RC (1992) Relationship between repetitive firing and afterhyperpolarizations in human neocortical neurons. J Neurophysiol 67:350-363.

Lorenzon NM, Foehring RC (1995) Characterization of pharmacologically identified voltage-gated calcium channel currents in acutely isolated rat neocortical neurons. I. Adult neurons. JNeurophysiol 73:1430-1442.

McCormick DA, Huguenard JR (1992) A model of the electrophysiological properties of thalamocortical relay neurons. J Neurophysiol. 68(4):1384-400

Nevian T, Larkum ME, Polsky A, Schiller J (2007) Properties of basal dendrites of layer 5 pyramidal neurons: a direct patch-clamp recording study. Nat Neurosci 10:206-214.

Schaefer AT, Helmstaedter M, Schmitt AC, Bar-Yehuda D, Almog M, Ben-Porat H, Sakmann B, Korngreen A (2007) Dendritic voltage-gated K+ conductance gradient in pyramidal neurones of neocortical layer 5B from rats. J Physiol 579:737-752.

Sidiropoulou K, Poirazi P (2012) Predictive features of perstistent activity emergence in regular spiking and intrinsic bursting model neurons. PLOS Comp Biol 8(4): e1002489
